# Current Thoughts

**Published:** 1900-01

**Authors:** 


### CURRENT THOUGHTS.

A WELL-KNOWN authority on livestock—a judge and breeder of national reputation—while not particularly antagonistic to the tuberculin test, said very emphatically in a recent interview:

"I can go into a herd of Jerseys, or Holsteins, or Shorthorns, or any other breed, and pick out by physical examination almost every cow that has tuberculosis. They are bound to show the presence of the disease if it has progressed far enough to be dangerous."—*Jersey Bulletin.*

While we know there are men possessed with great talent, yet this assertion will need some further explanation before we discard tuberculin and go back to physical examination alone. What is meant by "almost every cow?" Does he mean a large percentage of errors? Then again he says, "They are bound to show the presence of the disease if it has progressed far enough to be dangerous." Now, will he explain what symptoms or signs will lead us to know when the danger line has occurred? If he can do this we recognize the fact that he has no use for tuberculin. We know that our best and highest educated veterinarians make the assertion that tuberculin is the most sensitive and exact method of making a diagnosis of tuberculosis that we possess to-day.

We find in our practice that the dairyman judges us by our exactness.

Unless this gentleman can teach us his method of examination and prove to us that his percentage of errors will compare favorably with tuberculin, we will continue to use tuberculin. But when we use good judgment and physical examination with the tuberculin test we will make but few errors.

